# CREB3 subfamily transcription factors are not created equal: Recent insights from global analyses and animal models

**DOI:** 10.1186/2045-3701-1-6

**Published:** 2011-02-17

**Authors:** Chi-Ping Chan, Kin-Hang Kok, Dong-Yan Jin

**Affiliations:** 1 Department of Biochemistry and State Key Laboratory for Liver Research, LKS Faculty of Medicine, The University of Hong Kong

## Abstract

The CREB3 subfamily of membrane-bound bZIP transcription factors has five members in mammals known as CREB3 and CREB3L1-L4. One current model suggests that CREB3 subfamily transcription factors are similar to ATF6 in regulated intramembrane proteolysis and transcriptional activation. Particularly, they were all thought to be proteolytically activated in response to endoplasmic reticulum (ER) stress to stimulate genes that are involved in unfolded protein response (UPR). Although the physiological inducers of their proteolytic activation remain to be identified, recent findings from microarray analyses, RNAi screens and gene knockouts not only demonstrated their critical roles in regulating development, metabolism, secretion, survival and tumorigenesis, but also revealed cell type-specific patterns in the activation of their target genes. Members of the CREB3 subfamily show differential activity despite their structural similarity. The spectrum of their biological function expands beyond ER stress and UPR. Further analyses are required to elucidate the mechanism of their proteolytic activation and the molecular basis of their target recognition.

## Introduction

**Figure 1:**
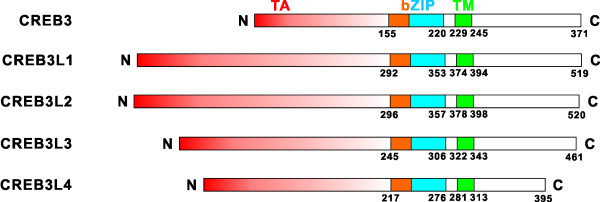
**The human CREB3 subfamily of transcription factors**. The bZIP domains of the members are aligned. TA: transcriptional activation domain. bZIP: basic leucine zipper domain. TM: transmembrane domain.

**Figure 2:**
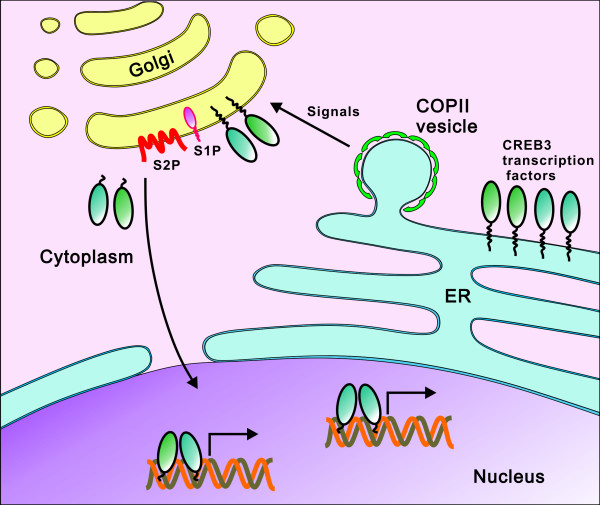
**Activation of membrane-bound transcription factors of the CREB3 subfamily**. CREB3 subfamily proteins are anchored to the ER membrane in an inactive form when they are not stimulated. Only upon stimulation, the CREB3 subfamily proteins are translocated by COPII vesicles from the ER to the Golgi apparatus where they will encounter S1P and S2P proteases. They are cleaved to release the N-terminal fragments which will enter the nucleus to activate transcription of target genes. CREB3 subfamily proteins might work as homodimers or heterodimers in the nucleus to activate gene expression.

The CREB3 subfamily of bZIP transcription factors in mammals comprises CREB3 (also known as LZIP or Luman), CREB3L1 (OASIS), CREB3L2 (BBF2H7), CREB3L3 (CREB-H) and CREB3L4 (AIbZIP) [1]. CREB3, the prototype of this subfamily, was first identified through its interaction with a transcriptional coactivator termed host cell factor 1 (HCF1) [2]. CREB3L1 was initially found in long-term cultured mouse astrocytes and thought to have a role in gliotic events [3]. CREB3L2 was identified as part of a fusion oncoprotein named FUS-CREB3L2, which was generated by a chromosomal translocation in low grade fibromyxoid sarcoma (LGFMS) [4]. For CREB3L3, it was first reported to be a liver-specific transcription factor [5]. CREB3L4 was originally identified as a highly expressed and androgen-induced protein in prostate cancer cells [6]. The CREB3 subfamily members are closely-related to *Drosophila *dCREB-A/BBF2 [7,8]. They share significant homology within their bZIP domain that mediates DNA-binding and dimerization [9]. Similar to ATF6, they also feature a transmembrane domain at the immediate C-terminal side of the bZIP region (Figure 1) [1,10]. As such, they are type II membrane-associated proteins with the N-terminus facing the cytoplasm and the C-terminus penetrating through the endoplasmic reticulum (ER) membrane into the ER lumen [11,12]. Based on their structural similarity to ATF6, transcription factors of the CREB3 subfamily are thought to be activated through regulated intramembrane proteolysis (RIP) in response to ER stress [12]. Upon activation, they are transported from the ER to the Golgi apparatus and proteolytically cleaved there by site 1 protease (S1P) and S2P sequentially to release the N-terminal fragment, which translocates into the nucleus to activate the transcription of genes that play important roles in unfolded protein response (UPR) [12,13]. This model is generally applicable to ATF6 and all CREB3 subfamily transcription factors (Figure 2). In this model, the ER-anchored uncleaved full-length form of the transcription factor remains inactive. Activation through RIP ensures a rapid and timely response to ER stress and UPR [1,10,14-16]. RIP is therefore a rate-limiting step in transcription factor activation. In addition to RIP, there are several other regulatory points. For example, the N-terminal active form of CREB3 subfamily transcription factors can form homo- and hetero-dimers with differential transcriptional activity [9]. Particularly, CREB3L3 was suggested to form a heterodimer with ATF6 to synergistically activate target genes [17]. Whether this heterodimer is thermodynamically stable and functional remains to be determined.

Although it is well accepted that the CREB3 subfamily transcription factors are activated through RIP, the jury is still out for several fundamentally important questions in the model that explains their activation. First, the physiological stimuli for proteolytic activation of discrete transcription factors in the CREB3 subfamily remain to be identified. Particularly, it will be of interest to clarify whether all these transcription factors are activated by ER stress. Second, the physiological targets of these transcription factors should be further characterized. Importantly, whether and how they activate UPR genes and other targets warrant additional analyses. Finally, the physiological roles of different transcription factors in this subfamily merit further investigations. Specifically, it will be of pivotal importance to determine how different factors execute their function in different tissues and cells. In this minireview, we will briefly discuss recent findings that advance our understanding in the above three areas. Collectively, the new evidence supports the notion that the five members in this subfamily have cell type-specific roles. Malfunction of these transcription factors is implicated in different diseases including cancer.

### Physiological roles of CREB3 subfamily transcription factors

One important insight of the CREB3 subfamily transcription factors was derived from *Creb3l1 *and *Creb3l2 *knockout mice [18]. Both CREB3L1 and CREB3L2 were previously implicated in UPR in astrocytes or glioma cells. They were shown to undergo RIP under ER stress, leading to the activation of Grp78 [15]. Although hippocampal pyramidal neurons in *Creb3L1*^-/- ^mice were slightly more susceptible to kainic acid, which also induces ER stress [19], the lack of either Grp78 reduction or strong neuronal phenotypes in the knockout mice raised significant concerns about the validity of the earlier data obtained from cultured glioma cells. In addition, one recent study indicated that expression of CREB3L1 in pancreatic β-cells did not induce Grp78 or other classical UPR genes, but boosted a subset of genes involved in the production of extracellular matrix proteins [20]. Thus, further analyses or re-analyses are required to resolve the discrepancies in the activation of UPR genes by CREB3L1 and CREB3L2.

The new findings from mouse model indicated the requirement of CREB3L1 for bone formation [18]. CREB3L1 was found to be abundantly expressed in osteoblasts. In the *Creb3l1 *knockout mice, severe osteopenia was observed as the result of a drop in type 1 collagen in the bone matrix together with a decrease in the osteoblast activity. These osteoblasts were found to possess abnormally enlarged rough ER in which a large amount of bone matrix proteins were accumulated. The cause for this phenotype in *Creb3l1*^-/- ^osteoblasts is still unclear but it could be due to the lack of activation of CREB3L1 target genes required for transport of bone matrix proteins from ER to Golgi. On the other hand, the type 1 collagen gene *Col1a1 *was identified as a direct target of CREB3L1 whose transactivation is achieved via an enhancer element in the osteoblast-specific *Col1a1 *promoter. CREB3L1 was essential for bone formation, plausibly by activating *Col1a1 *transcription and the secretion of bone matrix proteins [18]. This is the first evidence in support of a role for CREB3L1 in the secretory pathway.

Interestingly, a back-to-back paper on *Creb3l2 *knockout mice demonstrated a crucial role of CREB3L2 in chondrogenesis [21]. Sec23a, a coat protein complex II (COPII) component for transporting proteins from ER to Golgi, was shown to be a target of CREB3L2. The *Creb3l2^-/- ^*mice suffered from severe chondrodysplasia and had an immature chest cavity causing death from suffocation within a short time after birth. There were defects in cartilage formation and the abundance of extracellular matrix proteins was prominently reduced. Abnormally enlarged ER, which contains aggregated type II collagen (Col2) and cartilage oligomeric matrix protein (COMP), was observed in the proliferating chondrocytes. These findings supported the model in which CREB3L2 activates the Sec23a-dependent pathway required for secretion of collagens and other extracellular matrix proteins during normal chondrogenesis [21]. This is in keeping with the role of CREB3L1 in bone formation [18]. Together, the two studies pointed to an essential role of CREB3L1 and CREB3L2 in protein secretion. This idea was further supported by two recent studies conducted in other model organisms. In one study, *Xenopus *CREB3L2 was shown to be required for activation of the secretory pathway during notochord formation [22]. In another more recent report, *Drosophila *dCREB-A as well as mammalian CREB3L1 and CREB3L2 were found to be major and direct regulators of secretory capacity [23].

CREB3L1 and CREB3L2 are more closely related to each other than to CREB3, CREB3L3 and CREB3L4. The data obtained from animal models raised two possibilities concerning the role of CREB3 subfamily transcription factors in the regulation of protein secretion. In one perspective, CREB3L1 and CREB3L2 might serve a specialized regulatory function in secretion, which is evolutionarily conserved in dCREB-A, the single CREB3-like protein in *Drosophila *[23]. In another model, all five members in the CREB3 subfamily including CREB3, CREB3L3 and CREB3L4 could be influential in the regulation of protein secretion in other cell types. Further investigations are required to clarify these two possibilities.

Consistent with a possible role of CREB3L3 in cell secretion, *Creb3l3*-knockdown and *Creb3l3*^-/- ^mice were found to have a defect in the mobilization of acute phase response upon ER stress [17]. CREB3L3 is highly expressed during hepatogenesis. However, neither *Creb3l3*-knockdown nor *Creb3l3*^-/- ^mice showed defects in liver formation [17]. In addition to acute phase response genes such as the ones encoding C-reactive protein and amyloid P-component, several other important genes such as those of hepcidin, phosphoenolpyruvate carboxykinase and glucose 6 phosphatase were also direct targets of CREB3L3 [24,25]. Thus, the physiological function of CREB3L3 extends to iron homeostasis, innate immunity and gluconeogenesis.

Knockout mouse models for *Creb3l4 *were created by two independent groups. The *Creb3l4*-deficient mice generated by one group had significantly reduced number of spermatozoa in the epididymis although their fertility was not affected [26]. This observation might be explained by increased apoptosis of meiotic or postmeiotic germ cells [26]. This was also confirmed in the *Creb3l4^-/- ^*mice made by the other group [27]. Although CREB3 is the prototype of the CREB3 subfamily, a knockout mouse model for CREB3 has not been reported. The physiological roles of CREB3 were deduced primarily through overexpression studies conducted in cultured cells and CREB3 interaction partners identified from yeast two hybrid analyses. Particularly, CREB3 was reported to interact with transcriptional coactivator HCF1 [26], hepatitis C virus core protein [28], CC chemokine receptor 1 [29] and dendritic cell specific transmembrane protein [30]. Whether CREB3 indeed plays a role in chemokine signaling and dendritic cell maturation *in vivo *awaits the creation of knockout mice.

### Physiological activators and targets of CREB3 subfamily transcription factors

Phenotypic characterization of gene knockdown and knockout mice has not only derived novel insights into the physiological roles of the CREB3 subfamily of transcription factors as detailed above, but also provided important clues to their activators and targets. Particularly, identification of their target genes in knockdown and knockout mice by use of microarray technology and other global approaches has substantially advanced our understanding of this issue. Because some of these activators and targets are cell type-specific, we should take the expression pattern of CREB3 subfamily transcription factors into consideration. Although all proteins in this subfamily have similar domain architecture, they exhibit quite different expression profiles in different tissues: CREB3 is ubiquitous [2]; CREB3L1 is more abundantly expressed in some tissues including pancreas, prostate and bone [18,31]; CREB3L2 can be detected in various tissues and the strongest expression occurs in placenta, lung, spleen, intestine and cartilage [4,21]; CREB3L3 has a liver specific expression [5,17,32]; and CREB3L4 is expressed in different organs such as pancreas, liver, and gonads, yet it is most abundant in prostate epithelial cells [33,34]. Thus it is understandable that CREB3 subfamily transcription factors might serve different physiological roles in their target cells by modulating distinct subsets of genes in response to specific stimuli.

Whether ER stress induces proteolytic activation of CREB3 subfamily transcription factors remains controversial. Earlier reports by independent groups suggested that they are proteolytically activated by UPR induced by pharmaceutical agents such as tunicamycin or thapsigargin [17,18,21]. On the contrary, no activation of CREB3, CREB3L3 and CREB3L4 was found upon ER stress in several other reports. For example, proteolysis of CREB3 was not seen when UPR is activated [35,36]. The same trend also applies to the proteolytic activation of CREB3L3 and CREB3L4, which could not be induced by tunicamycin or other ER stressors [33,37]. What would be the direct inducer of the proteolytic activation of CREB3 subfamily transcription factors if ER stress is not? There are three schools of thoughts on this issue. First, some membrane-associated bZIP proteins in plants can be activated by salt stress or heat stress [38,39]. From an evolutionary point of view, it will be of interest to see whether mammalian transcription factors of the CREB3 subfamily might also be responsive to similar types of cellular stress. Second, a prototypic membrane-bound transcription factor known as sterol regulatory element binding protein (SREBP) responds to intracellular concentration of cholesterol through an ER-anchored escort protein named SREBP cleavage-activating protein (SCAP) and its regulator Insig [40,41]. Further investigations are required to determine whether similar or distinct escort proteins might also be influential in the activation of CREB3 subfamily transcription factors. Third, the phenotypes of knockout mice and the targets of CREB3 subfamily transcription factors found by global analyses are most revealing in the identification of their physiological activators. For instance, because CREB3L1 and CREB3L2 appear to be critical regulators of protein secretion and are required for bone and cartilage formation [18,20-23], they are plausibly activated by physiological inducers of extracellular matrix protein secretion such as transforming growth factor β [42]. Likewise, proteolysis of CREB3L3, which plays a role in acute phase response, was found to be induced by interleukin 1β and interleukin 6 [17,43], proinflammatory cytokines that regulate acute phase response. In line with its activation of genes involved in lipid metabolism [17], fatty acids were found to be activators of CREB3L3 [44,45]. For CREB3L4, it was found that androgens could induce its expression in a dose-dependent manner [6]. Consistent with a role in dendritic cell maturation, CREB3 was recently shown to be proteolytically activated after stimulation with LPS [30]. Generally, physiological activators of CREB3 subfamily transcription factors are more diverse and not limited to ER stress alone.

CREB3 subfamily transcription factors are thought to recognize cAMP-responsive element (CRE; TGACGTCA), box-B element (TACACGTAATC), ER stress responsive element II (ERSE-II; ATTGG-N-CCACG) and UPR element (UPRE; TGACGTGG) [2,11,33,46,47]. Whether different factors prefer a particular enhancer element has not been systematically investigated. Interestingly, microarray analyses revealed that distinct subsets of target genes are activated by CREB3L1, CREB3L2, CREB3L3 and CREB3L4 [17,18,20,21,23,33]. For example, CREB3L1 target genes include collagen genes *Col1a1 *and *Col1a2 *as well as other genes implicated in matrix protein production such as *Opn*, *Ocn*, *Papss2*, *Matn1 *and *Chst12 *[18,20]. CREB3L2 activates *Sec23a*, *Sec23b *and *Sec24c *genes that are involved in protein secretion [21], it could also turn on a subset of innate immunity-related genes such as MxA [23]. CREB3L3 stimulates *Sap *and *Crp *genes that are important in acute phase response, as well as other genes that may affect lipid metabolism [17]. Lastly, *ANG*, *ANKH*, *AQP9*, *ID2 *and *KDELR3 *genes that are implicated in various cellular processes include protein sorting are regulated by CREB3L4 [33]. Therefore, it will be of particularly great interest to determine whether the different spectra of target genes might be attributed to the cell type and/or the recognition sequence. Because the enhancer sequence recognized by CREB3 subfamily transcription factors has only been characterized in a very limited number of target genes, bioinformatic analysis and experimental validation of the recognition sequence in additional target genes should be performed to clarify whether these transcription factors might recognize other elements. Although some of these target genes might still be pertinent to ER stress and UPR, many are directly involved in other biological processes such as protein secretion, acute phase response, innate immunity and lipid metabolism [17,18,20,21,23,33]. While elucidation of the link between ER stress and these processes would expand our knowledge concerning the biological function of UPR, full characterization of these targets might derive novel insight into the physiological roles of CREB3 subfamily transcription factors beyond ER stress and UPR.

### Roles of CREB3 subfamily transcription factors in cellular transformation

CREB3 subfamily transcription factors exist ambiently in an inactive form bound to the ER membrane. The active form is usually short-lived and rapidly degraded by the proteosome [32,37]. Plausibly, constitutive activation of these transcription factors might have deleterious effects that are either tumor suppressive or oncogenic in different contexts. For example, whereas the proapoptotic activity might suppress oncogenesis, aberrant stimulation of extracellular matrix protein secretion could promote tumor formation and metastasis. It is therefore not surprising that all five members of the CREB3 subfamily have been implicated in cellular transformation. Below we will briefly review the roles of individual members in different types of cancer.

CREB3 was known to be a binding partner of hepatitis C virus core protein. This interaction with the viral oncoprotein might interfere with a tumor suppressive function of CREB3 to promote cellular transformation in hepatocellular carcinoma [28]. CREB3 was also reported to be influential in the metastasis of breast cancer cells [48]. CREB3L1 and CREB3L2 are fusion partners of FUS in the generation of chimeric oncoprotein by chromosomal translocation in a soft tissue sarcoma known as LGFMS [1,4,49,50]. The FUS-CREB3L1 or FUS-CREB3L2 fusion oncoproteins include the N-terminal fragment of FUS and the C-terminal fragment of CREB3L1 or CREB3L2 harboring the bZIP domain [4]. FUS-CREB3L2 has a very high incidence of 96% in successfully evaluated LGFMS cases whereas FUS-CREB3L1 was only found in one exceptional case of LGFMS [49]. Since expression of FUS-CREB3L2 is under the control of the strong FUS promoter, FUS-CREB3L2 is generally thought to be overexpressed in LGFMS. Additionally, the N-terminal part of FUS also contributes transactivating and oncogenic properties as in the case of other FUS-containing fusion oncoproteins [51]. Thus, FUS-CREB3L2 is a hyperactive transcription factor that turns on the expression of various target genes. Consistent with a role of CREB3L2 in stimulating the secretion of extracellular matrix proteins [18,23], aberrant accumulation of collagens was observed in LGFMS [52]. In this regard, it will be of great interest to elucidate how overproduction of collagen might underlie the pathology of LGFMS. In further support of the oncogenic property of CREB3L2, it was also found to be involved in another chromosomal translocation in thyroid carcinoma that gives rise to the CREB3L2-PPARγ fusion oncogene [53]. The endocrine function of the thyroid gland prompted us to the hypothesis that CREB3L2-PPARγ might also exert an impact on protein secretion in thyroid carcinoma. Further investigations are needed to test this hypothesis. Finally, a recent RNAi screen revealed a role for CREB3L2 in the survival pathway in malignant glioma cells. This was mediated through ATF5 and an ATF5 target known as myeloid cell leukemia sequence-1 (MCL1). The transcription of ATF5 was activated by CREB3L2, which was induced by Ras-mitogen-activated protein kinase or phosphoinositide-3-kinase signaling pathway. Taken together, these findings revealed an antiapoptotic activity of CREB3L2 and provided a new mechanism to explain the uncontrolled proliferation of malignant glioma cells [54]. CREB3L3 was found to be underexpressed in hepatocellular carcinoma and to serve a growth suppressive role in hepatocytes [32]. In this regard, it will be intriguing to see whether the *Creb3l3*^-/- ^mice might be more susceptible to liver cancer. Finally, the higher abundance of CREB3L4 in prostate cancer cells and its inducibility by androgens are in line with a crucial role for CREB3L4 in prostate carcinogenesis [6,34]. Nevertheless, further elucidation of the roles of discrete transcription factors in the CREB3 subfamily in cellular transformation might derive new approaches for rational design of targeted anti-cancer agents.

## Conclusions

The CREB3 subfamily transcription factors are not only involved in ER stress and UPR as previously envisaged, but also play a crucial role in many other biological processes including cell secretion, bone and cartilage formation as well as oncogenesis. Understanding the physiological activators and targets of this subfamily by use of a combination of global unbiased analyses, individual gene approaches and animal models will derive new knowledge in transcriptional regulation and new strategies in the intervention of human diseases.

## Competing interests

The authors declare that they have no competing interests.

## Authors' contributions

CPC, KHK and DYJ wrote the review. All authors read and approved the final manuscript.
